# Combined Stimulation with the Tumor Necrosis Factor **α** and the Epidermal Growth Factor Promotes the Proliferation of Hepatocytes in Rat Liver Cultured Slices

**DOI:** 10.1155/2012/785786

**Published:** 2012-10-16

**Authors:** Francis Finot, Régis Masson, Fabienne Desmots, Catherine Ribault, Nicole Bichet, Joan A. Vericat, Patricia Lafouge, Christiane Guguen-Guillouzo, Pascal Loyer

**Affiliations:** ^1^Covance Laboratory SAS, 2-8 rue de Rouen, Z.I. de Limay-Porcheville, 78440 Porcheville, France; ^2^Laboratoire d'Hématologie, Hôpital Pontchaillou, 2 rue Henri Le Guilloux, 35033 Rennes Cedex, France; ^3^Inserm UMR-S 991, Université de Rennes 1, Hôpital Pontchaillou, 35033 Rennes Cedex, France; ^4^Noscira SA, 52 Avendia de la Industria 38760 Tres Cantos, Spain

## Abstract

The culture liver slices are mainly used to investigate drug metabolism and xenobiotic-mediated liver injuries while apoptosis and proliferation remain unexplored in this culture model. Here, we show a transient increase in LDH release and caspase activities indicating an ischemic injury during the slicing procedure. Then, caspase activities decrease and remain low in cultured slices demonstrating a low level of apoptosis. The slicing procedure is also associated with the G0/G1 transition of hepatocytes demonstrated by the activation of stress and proliferation signalling pathways including the ERK1/2 and JNK1/2/3 MAPKinases and the transient upregulation of c-fos. The cells further progress up to mid-G1 phase as indicated by the sequential induction of c-myc and p53 mRNA levels after the slicing procedure and at 24 h of culture, respectively. The stimulation by epidermal growth factor induces the ERK1/2 phosphorylation but fails to activate expression of late G1 and S phase markers such as cyclin D1 and Cdk1 indicating that hepatocytes are arrested in mid-G1 phase of the cell cycle. However, we found that combined stimulation by the proinflammatory cytokine tumor necrosis factor **α** and the epidermal growth factor promotes the commitment to DNA replication as observed *in vivo* during the liver regeneration.

## 1. Introduction

Isolation of hepatocytes from normal liver and establishment of *in vitro* culture systems have provided powerful experimental *in vitro* models to identify extracellular signals and to study intracellular signalling pathways regulating differentiation and controlling the ratio between proliferation and apoptosis in liver [1]. Enzymatic liver dissociation triggers G0/G1 transition of *in vivo*-quiescent hepatocytes, which progress up to and arrest in mid-G1 phase in absence of growth factors in primary culture [2]. In primary culture, expression of liver specific functions progressively decreases and apoptosis eventually occurs within a week through the activation of caspases 3, 8, and 9 in hepatocytes [3, 4]. Nevertheless, this *in vitro* culture model has been very useful to identify apoptotic inducers, survival factors and mitogens based on their ability to increase or reduce apoptosis and induce DNA replication, respectively. For instance, supplementation of culture medium with soluble factors such as insulin and glucocorticoids improves cell stability while epidermal growth factor induces cell proliferation [1].

More complex culture systems were further developed in which hepatocytes survive and remain differentiated for several weeks: (1) combination of additives to culture medium, including hormones, nicotinamide, phenobarbital, or dimethylsulfoxide (Me_2_SO) [5], (2) cocultures associating hepatocytes and nonparenchymal liver epithelial cells [6], (3) extracellular matrices such as Vitrogen [7], collagen type I, and Matrigel [8]. In Me_2_SO-treated cultures [9], coculture [10] and monolayers or sandwich of collagen I [11, 12], hepatocytes are arrested in G1 phase of the cell cycle and do not replicate DNA upon stimulation by growth factors while initiator caspases 8 and/or 9 are processed into cleaved mature forms but remain inactive, preventing maturation of terminal caspases, execution of apoptosis, and allowing longer survival of hepatocytes [4, 12]. 

An alternative model to the culture of isolated hepatocytes is the use of precision-cut liver slices [13]. A 250 *μ*m thick liver slice contains about ten intact cells layers maintaining normal tissue architecture with all liver cell types represented. This *in vitro* model is particularly suitable to evaluate selective intralobular hepatic toxicity of endogenous compounds [14, 15] and drugs [16–18], assess functional interaction between hepatocytes and nonparenchymal hepatic cells [19–21], study drug-induced hematologic disorders in whole blood cells cocultured with liver slices [22], and to study the mechanisms of HCV life cycle and new antiviral compounds [23]. Interest in liver slices as a drug evaluation system was reinforced by the demonstration that phase I and II enzymes were inducible by drugs [24, 25] and the establishment of cryopreservation methods [26–28]. As observed for isolated hepatocytes in primary culture, cultured slices progressively undergo a loss of cellular integrity evidenced by release of cytosolic enzymes [29], reduction in ATP content and decrease in expression of some of the liver specific functions [30, 31]. Optimization of slice preparation procedures [32, 33], culture conditions [34, 35], and culture devices [36, 37] with improved air-fluid interface for better cell oxygenation [38, 39] has allowed to significantly increase viability of adult liver slices for up to 10 days. 

 To the best of our knowledge, little is known about proliferation and apoptosis in cultured liver slices. It has been reported that a limited number of cells, mostly hepatic stellate cells [39], replicate DNA within the cultured slices [40]. However, it is still unknown whether hepatocytes remain quiescent or enter the cell cycle and can actively proliferate in cultured slices. Moreover, the expression and activity of caspases in liver slices during slicing procedure and in culture have not been studied yet.

In this report, we study the cell cycle entry and induction of apoptosis in hepatocytes in precision-cut slices. We demonstrate that liver slicing procedure induces proliferation signalling pathways, which trigger entry into and progression through G1 phase of the cell cycle similar to that observed in isolated hepatocytes after liver dissociation. In addition, hepatocytes in cultured slices undergo apoptosis at very low rates even after treatment with apoptotic factors TGF*β* and TNF*α*, and do not proliferate upon EGF stimulation suggesting that cell-cell and/or cell-ECM interactions protect from apoptosis and inhibit G1/S transition. However, costimulation by TNF*α* and EGF overrides this G1 phase arrest demonstrating that proliferation of hepatocytes can be induced in cultured rat liver slices by proinflammatory cytokines and growth factors as observed *in vivo* during liver regeneration.

## 2. Material and Methods

**Chemicals and Reagents**

Bovine serum albumin (BSA) fraction V (Boehringer, Mannheim biochemicals), recombinant human (rHu) epidermal growth factor (EGF, Promega), rHu TNFalpha (Promocell, Heidelberg, Gremany), transforming growth factor 1 (R and D Systems, Abingdon); bovine insulin, dimethyl sulfoxide (Me_2_SO), PIPES, CHAPS, orthovanadate, benzamide, aprotinin, leupeptin, and soybean trypsin inhibitor were purchased from Sigma Chemical Company (USA). Rediprime II DNA labelling kit, DNA herring sperm, [*α*-^32^P]dCTP (3000 Ci/mmol), and [H^3^]-methyl-thymidine (25 Ci/mmol) were purchased from Amersham Life Sciences. Dulbecco's modified Eagles medium (DMEM) with 4.5 mg/mL glucose and L-glutamine came from B.I. BioWithacker fetal calf serum was from Gibco BRL. The detection of cyclin D1, p53, Cdk4, c-fos, and c-myc mRNAs by Northern blotting was performed as previously reported [2].* Antibodies: *Anticaspase-3 (H-277, Santa-Cruz Biotechnology), anticaspase-8 (APP-108) and anticaspase-9 (APP-109) were from StressGen Biotechnologies Corp. (Tebu, France); antialbumin and -transferrin (Kent Laboratories, Redmond, WA, USA); CYP3A1/2 and CYP2B (Daiichi Pure Chemicals Co., Ltd., Tokyo, Japan); CYP2E1 (Oxford Biomedical, USA); GSTA1 and GSTP1 were from Biotrin (Dublin, Ireland); anti-Cdk1 and -GSTA4 antibodies were previously described [2, 41]; anti-cyclin D1 (Ab-3, Neomarkers); anti-phospho-JNK (sc6254) and total JNK (sc571) and HSC70 (sc7298) were from Santa-Cruz Biotechnology. Rabbit polyclonal anti-Phospho-Histone H3 (ser10), anti-STAT3 (#9132) and anti-phospho-STAT3 (Tyr705, #9131) were from Cell Signalling. The secondary antibodies conjugated to horseradish peroxidase were purchased from DAKO (France). Fluororimetric substrates Ac-DEVD-AMC, Ac-IETD-AMC, and Ac-LEHD-AMC were from BACHEM (BACHEM, Voisins-Le-Bretonneux). Supersignal came from Pierce Chemical Co. (Rockford, IL, USA).

**Animals**

Male Sprague-Dawley rats (13 weeks old) were obtained from IFFA CREDO (L'Arbresle, France). They were kept under controlled environmental conditions (12 hr light-dark cycle) and fed a standard diet (Animalabo A 04, water *ad libidum*). Procedures for housing the rats, isolation, and culture rat hepatocytes were in agreement with the French regulation.

**Preparation and Culture of Liver Slices**

The liver was rinsed *in situ* (20 mL/min) with cold oxygenated (95% O_2_ and 5% CO_2_, 0.4 L/min) Krebs-Henseleit Bicarbonate buffer, pH 7.4, for 4 to 6 min until the appearance of a homogenous brown color. The liver was then perfused with Viaspan (Belzer's University of Wisconsin solution, Dupont Pharma). Liver slices (250 *μ*m) were prepared according the method of Smith et al. [42], then, preincubated for 90 min in Waymouth medium (supplemented with 10% FCS, 100 IU/mL penicillin, 50 mg/mL streptomycin and 1.7 mM insulin) at 37°C in 95% O_2_ and 5% CO_2_ atmosphere (0.2 L/mL) in a dynamic culture system (Vitron Incubator). After the preincubation, culture medium was replaced by serum free medium.

**Isolation and Primary Culture of Hepatocytes**

Primary rat hepatocytes were isolated and purified from male Sprague-Dawley rats as described previously described [6]. Hepatocytes were seeded at 7·10^4^ cells/cm^2^ on plastic dishes in a mixture of 75% minimum essential medium and 25% medium 199, supplemented with 10% fetal calf serum (FCS), and per mL: 100 IU of penicillin, 100 *μ*g of streptomycin, 1 mg of bovine serum albumin (BSA), 2 *μ*mol L-glutamine, and 5 *μ*g of bovine insulin. Four hours after plating, the medium was removed and cultures were maintained in the same FCS free medium supplemented with 1.4·10^−6^ M hydrocortisone hemisuccinate (Roussel-Uclaf).

**Treatments with Apoptotic Inducers and Growth Factors**

In primary culture of isolated hepatocytes (6-well plates, 5·10^5^ cells per well), TGF*β*1 (2.5 ng/mL), cycloheximide (5 *μ*g/mL), TNF*α* (20 ng/mL), or both cycloheximide and TNF*α* began 24 hours after plating and were carried out for 24 (TNF*α* and/or cycloheximide) or 48 (TGF*β* and TNF*α*) hours. In cultured slices (~1.5·10^6^ cells/slice), concentrations of compounds were adjusted to obtain similar amounts per cell number. Treatments began at 4 hours and were carried out for 24 hours with TNF*α* (55 ng/mL) and/or cycloheximide (25 *μ*g/mL) or 48 hours with TGF*β* (15 ng/mL) and TNF*α*. For inducing proliferation, EGF (50 ng/mL for isolated hepatocytes and 250 ng/mL for cultured slices) was added during all the culture time.

**Biochemical Endpoints**

In the incubation medium collected at 3, 24, 48 and 96 hours, LDH contents were measured using Boehringer Mannheim MPR2 kit according manufacturer's instructions. LDH release was calculated with the ratio: extracellular LDH/total LDH (intra + extracellular). Intracellular ATP was measured by the luciferin/luciferase reaction using HSII kit (Roche) and a luminometer Microlumat-Plus EGG-Berthold.

**RNA Extraction and Northern Blot Analysis**

Liver slices were harvested and stored at −80°C. Total RNA was extracted using SVRNA extraction Kit from Qiagen (Courtaboeuf, France) and quantified by ultraviolet absorption at 260 nm. Integrity of the RNA samples was confirmed by formaldehyde agarose gel electrophoresis and visualisation by ethidium bromide staining of 18S and 28S ribosomal RNAs. The RNA samples (20 *μ*g) were resolved by electrophoresis in a 1% agarose gel containing 1.85% formaldehyde and transferred to a nylon membrane (Hybond-N+, Amersham Life Science, The Netherlands). Hybridization was carried out using ^32^P-labeled cDNA probes at 65°C overnight.

**Fluorimetric Caspase Activity Assay**

Liver slices and cultured hepatocytes were harvested and washed with PBS and lysed in the caspase activity buffer containing 20 mM piperazine-N,N′-bis-(2-ethanesulfonic acid) (PIPES) pH 7.2, 100 mM NaCl, 10 mM DTT, 1 mM EDTA, 0.1% 3-(3-cholamidopropyl-dimethylammonio)-2-hydroxy-1-propanesulfonic acid (CHAPS), 10% sucrose as previously described [4]. 100 *μ*g of crude cell lysates were incubated with 80 *μ*M substrate-AMC at 37°C for 1 hour. Caspase mediated cleavage of peptide-AMC was measured by spectrofluorometry (Molecular Device) at the excitation/emission wavelength pair (ex/em) of 380/440 nm. The caspase activity was presented in arbitrary units of fluorescence (per 100 *μ*g of total proteins).

**Histology and Immunostaining of Ki67**

For semi-thin sections, liver slices were collected, fixed with 25% glutaraldehyde in 0.4 M Cacodylate buffer, pH 7.2), postfixed in 2% osmium tetroxide and embedded in Epon-Araldite resin. Semithin section (1 *μ*m) were cut, stained with azur blue and examined with a Leitz DMRB light microscope. Hematoxylin and eosin staining was performed on paraffin sections while the immunodetection of Ki67 was performed on frozen sections (Histopathology H2P2 core facility, Fédération de Recherche, Biosit, University of Rennes 1).

**Immunoblotting Analysis**

Liver slices and cultured hepatocytes were lysed by sonication in lysis buffer containing 50 mM HEPES pH 7.5, 150 mM NaCl, 15 mM MgCl_2_, 1 mM EDTA, 2.5 mM EGTA, 1 mM DTT, 0.1% Tween 20%, 0.1 mM sodium orthovanadate, 1 mM NaF, 10 mM *β*-glycerophosphate, 0.1 mM phenylmethylsulfonyl fluoride, 100 *μ*g/mL benzamidine and 5 *μ*g/mL aprotinin, leupeptin, and soybean trypsin inhibitor. Protein concentrations were quantified using the Biorad protein assay (Bio-Rad, France). 100 *μ*g of proteins were resolved on SDS-PAGE and transferred onto polyvinylidene difluoride membranes (PVDF, Biorad). Nonspecific binding sites were blocked with Tris Buffer Saline (TBS) containing 4% BSA, for 1 hour at room temperature. Then, filters were incubated overnight at 4°C with primary antibody diluted at 1 : 250 for anticaspase 3, 1 : 1500 for anticaspase 8, 1 : 600 for anticaspase 9, and 1 : 2000 for other antibodies in TBS containing 4% BSA. Filters were washed three times with TBS and incubated with appropriate secondary antibody conjugated to horseradish peroxidase, for 1 hour at room temperature. Proteins were visualized with Supersignal (Pierce Chemical Co., Rockford, IL).

**Statistics**

The data presented in this manuscript were obtained from 3 to 6 independent experiments. In each experiment, one rat was killed and all the slices were prepared from the same liver. For each time point 1 to 3 slices were used. For each condition and time-point, the experiment was repeated 2 to 6 times. In figure legend, detailed information was given on the number of experiments performed. Results in tables and figures are expressed as mean ± SD. In some experiments, statistical significance between control and treated hepatocytes was tested by a paired Student's *t*-test. A *P* value of < 0.05 was considered to be statistically significant.

## 3. Results

### 3.1. Viability and Expression of Specific Liver Functions in Cultured Liver Slices

**Figure 1:**
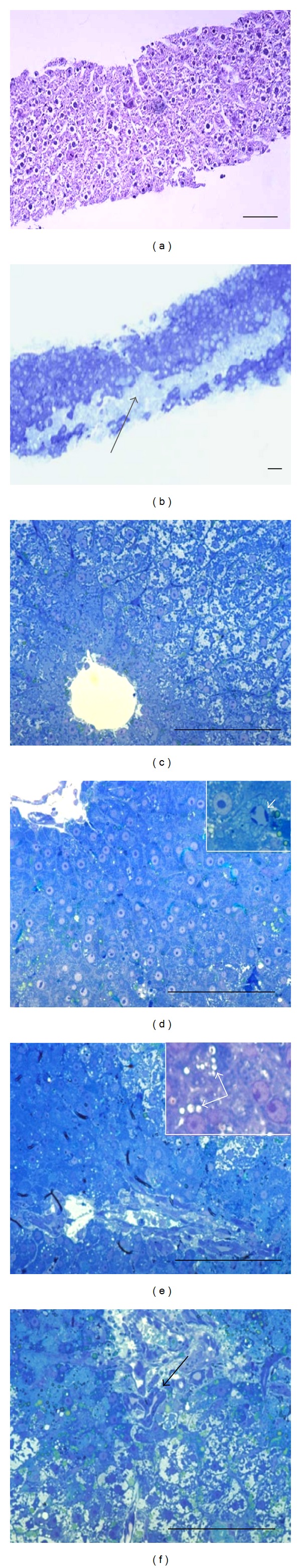
Histology of liver slices in culture. Histology of transversal (a, b) and longitudinal sections (c–f) of rat liver slices immediately after preparation (a) and after 24 (b, c), 48 (d), 72 (e), and 96 h (f) of culture. Hematoxylin and eosin staining illustrates the integrity of liver architecture after slicing (a) and the appearance of a thin necrotic area at the center of the slice ((b), dark arrow). (c–f), semithin sections stained with azur blue evidenced the apoptotic bodies at 48 h ((d), white arrow), the microvacuoles in hepatocytes at 72 h ((e), white arrow) and disorganized periportal zone at 96 h ((f), dark arrow); bar 100 *μ*m.

**Figure 2:**
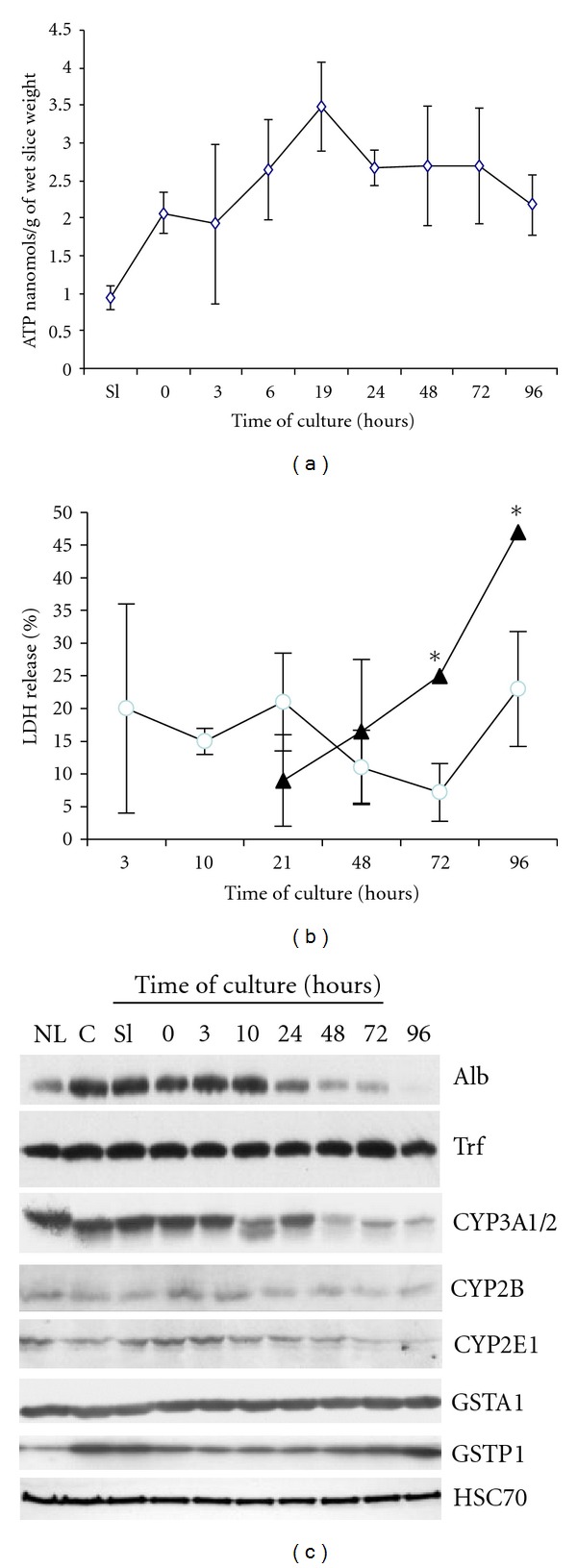
ATP content, LDH release, and expression of liver specific proteins. ATP content, expressed in nanomols/g of wet slices weight (a) and LDH release (b) were measured during the preincubation (Sl) and at the indicated times of culture. LDH release was also measured in primary culture of isolated hepatocytes (dark triangles). Western blot analysis of liver specific proteins (c) in normal liver (NL), in core before slicing (c), freshly prepared slice (Sl), after the preincubation (T0), and in culture at different times. Specific antibodies detecting albumin (Alb), transferrin (Trf), cytochrome P450 (CYP) 3A1/2, 2B, 2E1 subunits, and glutathione S-transferases (GST) A1 and P1 isoforms, were used. The western blot of HSC70 indicated that equal amounts of proteins were loaded in each lane. These experiments were performed on 2 independent experiments with 3 slices in each experiment. *P < 0.001 treatment versus control.

**Figure 3:**
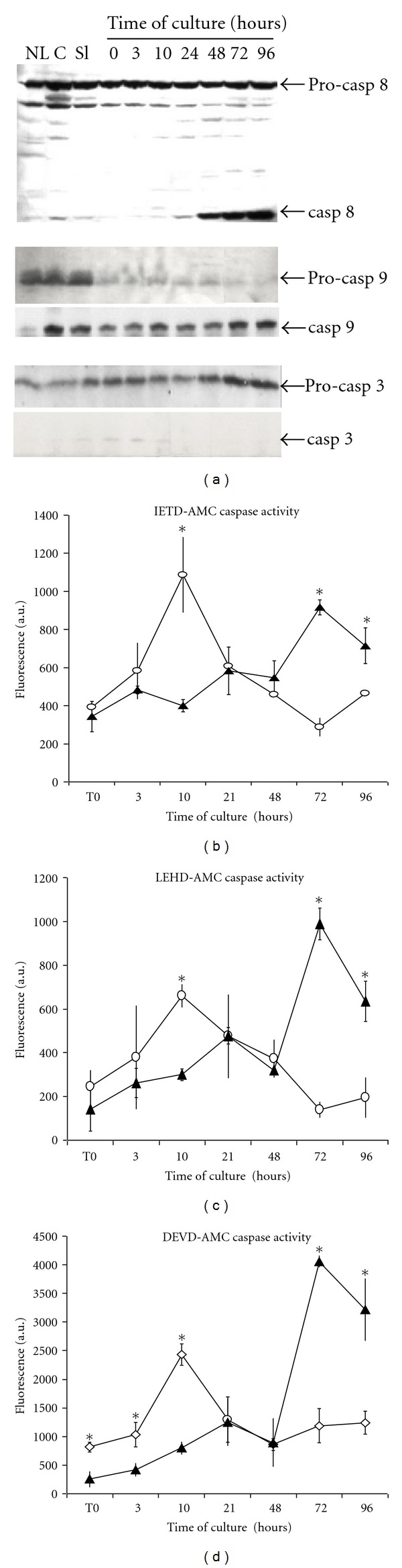
Time course of caspase expression and activation. (a), Western blot analysis of caspase 8, 9 and 3 in normal liver (NL), core (C), after slicing (Sl), after pre-incubation (T0) and at the indicated times of culture. IETD- (b), LEHD- (c) and DEVD-AMC (d) caspase activities were measured in cell lysates from slices (open circles) and isolated hepatocytes (dark triangle) at different times of culture. Activities were expressed in arbitrary units (A.U.) of fluorescence. Caspase activities in isolated hepatocytes were measured in 6 independent experiments while activities in liver slices are the results of 3 independent experiments with 2 or 3 slices in each experiment. *P < 0.001 treatment versus control.

In order to evaluate cell viability of cultured liver slices, histological integrity was studied (Figure 1) and correlated with ATP content, LDH release, and expression of specific liver functions (Figure 2). Histology of freshly prepared slices after a 90 min preincubation in medium, indicated a normal liver architecture despite a few damaged cells and dilated sinusoids on the edge of the slices (Figure 1(a)). After 24 hours (h) of culture, the slices exhibited normal liver histology although a necrotic zone restricted to 1 or 2 layers of hepatocytes at or near the center of the slice, could be observed (Figure 1(b)). In viable midzone areas, hepatocyte clarification corresponding to glycogen content eliminated during the fixation and dehydration steps of semi-thin section preparation, was clearly evidenced (Figure 1(c)). At 48 h of culture, scattered hepatocytes with microvacuoles were detected while the glycogen content was markedly reduced (Figure 1(d)). In addition, apoptotic cells, characterized by nuclear chromatin condensation and formation of apoptotic bodies, were observed (Figure 1(d), inset). At 72 h, hepatocytes located mostly in periportal area and in a lesser extent in midzonal area exhibited lucent microvacuoles containing dense material (Figure 1(e), inset). Apoptotic figures were no longer detected. At 96 h, light swollen and dark hepatocytes were detected in disorganised cords in periportal zone indicating that necrotic areas had significantly enlarged (Figure 1(f)). The ATP content (Figure 2(a)) was low in freshly prepared slices (1 nmol/g of slice) reflecting a low energy charge immediately after slicing. This content strongly increased during the preincubation step and in culture up to 19 h after plating to reach 3.5 nmol/g and, then, remained stable until 96 h. LDH release was measured in primary culture of isolated hepatocytes and cultured liver slices. In isolated hepatocytes, LDH release is relatively low during the first days of culture but increases with time to reach very high levels at 96 hours concomitantly with the strong increase in caspase activities (Figure 3). In liver slices, the LDH release was consistently high during the first hours of culture (3 to 24 h), decreased at 48 and 72 h of culture and increased again at 96 h (Figure 2(b)).

Expression of several liver specific proteins including albumin and transferrin, phase I enzymes cytochromes P450 (CYP) 3A1/2, 2E1 and 2B and phase II enzymes glutathione S-transferases (GST) A1 and P1, was analyzed by western blot (Figure 2(c)). Protein levels of these liver specific functions were similar in liver, core, freshly prepared slices and cultured slices up to 3 to 10 hours of culture. Then, three groups of functions could be distinguished: in the first one, levels of albumin and CYP3A1/2 and 2E1 progressively decreased with time; in the second group, the expression of transferrine, CYP2B and GSTA1 were maintained compared to normal liver, without significant changes during 4 days. Finally, the expression of GSTP1 was higher in core and freshly prepared slices compared to normal liver, then decreased between 3 and 48 h before increasing again at 72 and 96 h.

### 3.2. Early and Transient Induction of Caspase Activities in Cultured Liver Slices

In primary culture, isolated hepatocytes undergo apoptosis within 4 days through the activation of caspases 3, 8, and 9, [3, 4]. To determine whether apoptosis also took place in cultured liver slices, western blot analysis were performed to evidence both the pro- and cleaved forms of the initiator caspases 8 and 9 and the executioner caspase 3 (Figure 3(a)). 

No changes in the level of procaspase 8 were observed during the 4 days of culture while the cleaved form of this initiator caspase, undetectable in liver and cultured slices at 3, 10, and 24 h, appeared at 48 h and increased with the time of culture thereafter. Levels of procaspase 9 progressively decreased and became very low at 96 hours while the cleaved product was immediately detected after slicing and remained present during 4 days. The expression of procaspase 3 progressively increased with culture time. Very low amounts of cleaved caspase 3 were evidenced in freshly prepared slices and during the first 10 to 24 h of culture but not thereafter (Figure 3(a)). 

 To determine whether the cleaved form of caspases detected in cultured liver slices were active, caspase 8, 9, and 3 activities were measured using their main fluorogenic tetrapeptide substrates IETD-, LEHD, and DEVD-AMC, respectively (Figures 3(b)–3(d)). These caspase activities were measured in cell lysates from freshly prepared and cultured slices at different times and compared to activities in isolated and cultured hepatocytes. All three activities were very low in freshly prepared slices and isolated hepatocytes although DEVD-AMC was slightly higher in liver slices than in isolated hepatocytes. During the first 10 h of culture, caspase activities sharply increased in slices but not in isolated cultured hepatocytes, then, decreased at 21 and 48 hours to reach the values measured in cultured hepatocytes. A strong induction of these caspase activities was observed at 3 and 4 days in cultured hepatocytes as previously reported [4] but not in liver slices.

**Figure 4:**
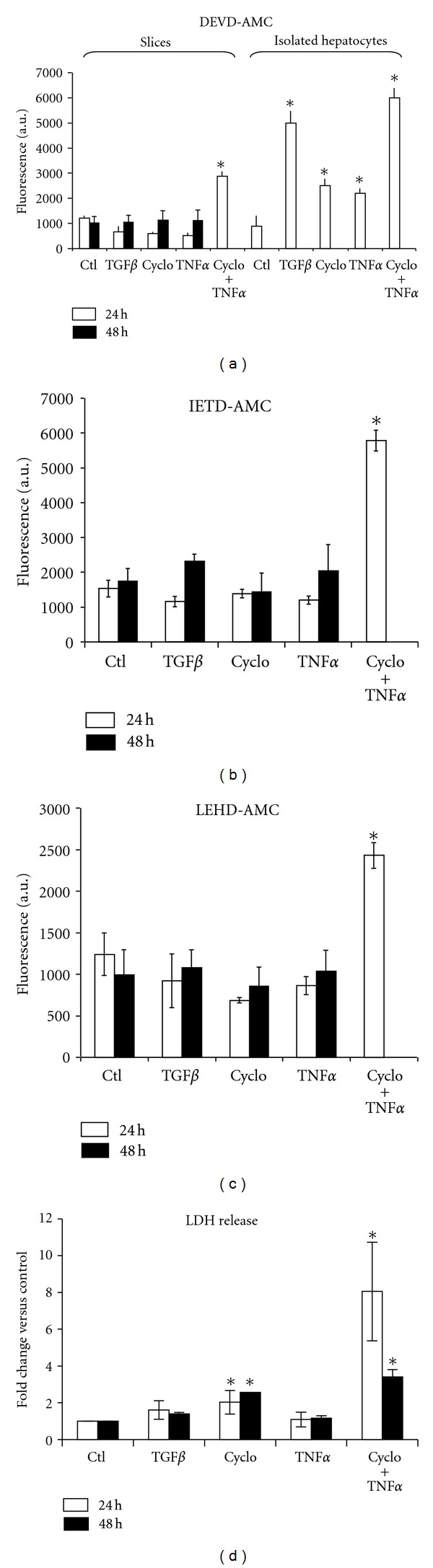
Caspase activities and LDH release in cultured slices and hepatocytes upon treatments with TGF*β*, TNF*α*, and cycloheximide. DEVD- (a), IETD- (b), LEHD-AMC (c), caspase activities, and LDH release (d) in cultured slices at 24 and 48 h in control (Ctl) after treatments with TGF*β*, cycloheximide, TNF*α*, and TNF*α* + cycloheximide. For slices treated with both TNF*α* and cycloheximide, caspase activities at 48 h were not presented because of a complete loss of viability between 24 and 48 h. Caspase activities and LDH release in liver slices are the results of 3 independent experiments with 2 or 3 slices in each experiment. *P < 0.001 treatment versus control.

 We then compared the induction of apoptosis by treatments with the apoptotic factors TGF*β*, cycloheximide, TNF*α*, and TNF*α* plus cycloheximide in cultured liver slices and primary culture of isolated hepatocytes (Figure 4). In liver slices, neither TGF*β*, TNF*α*, nor cycloheximide increased the DEVD- (Figure 4(a)), IETD- (Figure 4(b)), and LEHD-AMC (Figure 4(c)) caspase activities while the cotreatment with cycloheximide and TNF*α* led to a strong induction of these activities. In contrast, in pure culture of hepatocytes, all four treatments strongly induced caspase activities. In addition, LDH release was also studied in cultured slices to confirm that these treatments did not affect cell viability (Figure 4(d)). As observed with caspase activities, cycloheximide combined to TNF*α* led to a strong LDH release. TGF*β* and TNF*α* alone did not induce cell death while cycloheximide triggered a moderate but significant LDH release without detectable induction of caspase activities.

### 3.3. Hepatocytes in Slices Enter into and Progress through G1 Phase of the Cell Cycle

**Figure 5:**
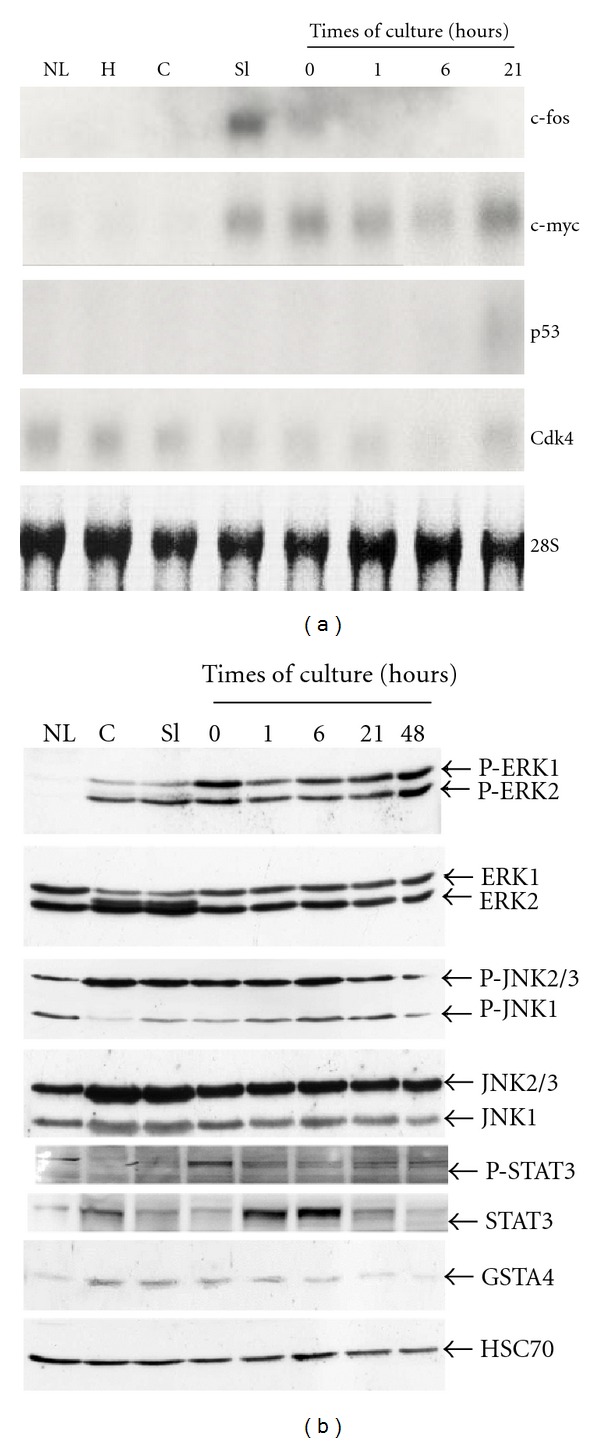
Sequential activation of cell cycle markers and signalling pathways.(a) Northern blot analysis of c-fos, c-myc, and p53 mRNAs. Samples were: livers collected after perfusion by Krebbs buffer (L) and Viaspan solution (H), core (C), freshly cut slices (Sl), after preincubation (0), and at 1, 6, and 21 h of culture. Cdk4, known to be expressed in normal liver and throughout the cell cycle, and 28S ribosomal RNA were used to control equal loading of RNAs in each lane. (b) Western blot analysis of phospho- and total-ERK1/2, STAT3, and -JNK1/2/3. GSTA4, a marker of oxidative stress, was also studied while HSC70 was used as loading control. These data were found similar in 2 independent experiments.

To determine whether cells in liver slices remained quiescent in G0 or entered the G1 phase of the cell cycle, we analyzed, by northern blotting, the levels of the protooncogenes c-fos and c-myc mRNAs (Figure 5(a)), two hallmarks of G0/G1 transition and early G1 phase [43], respectively, during the slicing procedure and in culture. Neither c-fos nor c-myc mRNAs were detectable in liver after *in situ* liver perfusion or in liver core but were strongly induced in freshly prepared slices. In culture, c-fos mRNA levels rapidly decreased indicating a very transient expression while c-myc expression was stable for at least 21 h of culture. We then investigated the expression of the tumour suppressor gene p53 mRNA, a mid-G1 phase marker. In liver, core, and slices during the first 10 h of culture, p53 mRNAs were not detected. A late induction was found at 21 h. Cdk4 mRNAs, known to be expressed in normal liver and throughout the cell cycle, were detected in all samples with little changes in the expression levels. These results demonstrated that cells in liver slices expressed markers of early and mid-G1 immediately after slicing strongly suggesting G0/G1 transition and progression in early G1 phase of cells in cultured liver slices.

 G0/G1 transition is controlled by cytokines and oxidative stress activating intracellular signalling pathways including MEK/ERK, STAT3, and JNK [44, 45]. In order to determine if these pathways were activated in liver slices, we investigated by immunoblotting expression of phosphorylated or total forms of ERK1/2, STAT3, and JNK1/2/3 as well as the GSTA4 (Figure 5(b)), a GST isoform induced by and involved in metabolism of lipid peroxidation products [41, 46]. Phospho-ERK1/2 and -JNK1/2/3 were strongly induced immediately after liver perfusion and were maintained during at least 48 h of culture demonstrating the early and robust activation of these two signalling pathways. Total STAT3 was detected in all slice extracts but was strongly induced at 1 and 6 hours of culture. Its phosphorylated form was expressed at a low level in normal liver, undetected during the slicing procedure but was induced in cultured slices. GSTA4 was also induced in core and freshly prepared slices but its expression level slowly decreased with time of culture. 

 Taken together, these data demonstrate the rapid activation of MAPKinase pathways during slicing and induction of downstream genes involved in proliferation such as c-fos, c-myc, and p53 in cultured slices strongly suggesting the entry into and progression through early G1 phase of cells in livers slices. 

### 3.4. G1/S Transition Requires Costimulation by EGF and TNF*α* in Cultured Liver Slices

In conventional cultures of isolated hepatocytes stimulation by growth factors such as EGF, TGF*α* and HGF triggers the G1/S transition [47]. 

**Figure 6:**
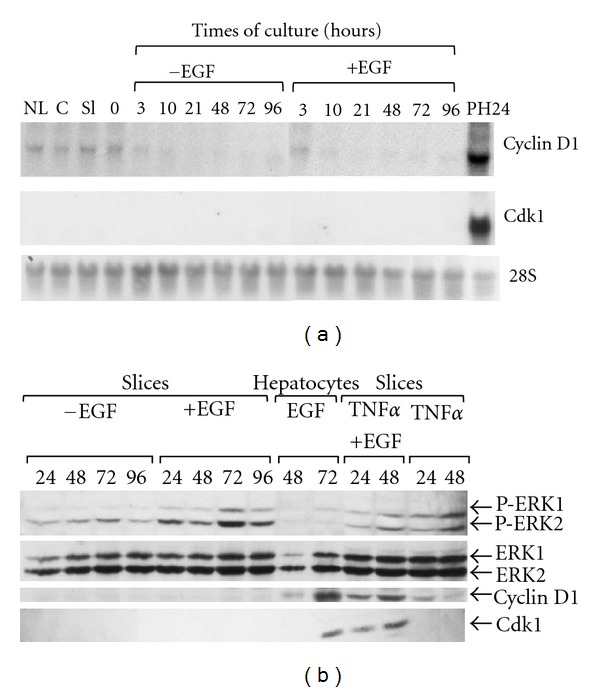
Expression of cyclin D1, Cdk1, and ERK1/2. Northern blot analysis of cyclin D1 and Cdk1 (a) in liver (NL), core (c), freshly prepared slices (Sl), after preincubation (0), and at different times of culture in absence (−EGF) or presence (+EGF) of EGF. Regenerating liver, 24 h after partial hepatectomy (PH24), was used as a positive control of proliferation. Hybridization of 28S ribosomal RNAs was used to control equal loading of RNAs in each lane. Western blot analysis of phospho-(P-)ERK1/2, total ERK1/2, cyclin D1, and Cdk1 (b) in cultured slices at the indicated times of culture in absence (−EGF) or presence (+EGF) of EGF and/or TNF*α*. Primary cultures of isolated hepatocytes stimulated by EGF (at 48 and 72 h) were used as control of proliferation.

To determine whether cells in cultured slices replicated after stimulation by growth factors, slices were maintained in culture for 4 days in absence or presence of EGF and mRNA levels of cyclin D1 and cdk1 known to be induced in late G1 and S phases, respectively [48], were analyzed (Figure 6(a)). Cyclin D1 mRNAs were detected at low levels in liver tissue and during slicing and were undetectable in cultured slices in absence or presence of EGF. Cdk1 mRNAs were never detected in any slice samples. In contrast, cyclin D1 and cdk1 were strongly induced in regenerating liver 24 h post-hepatectomy, used as positive control of proliferation.

**Figure 7:**
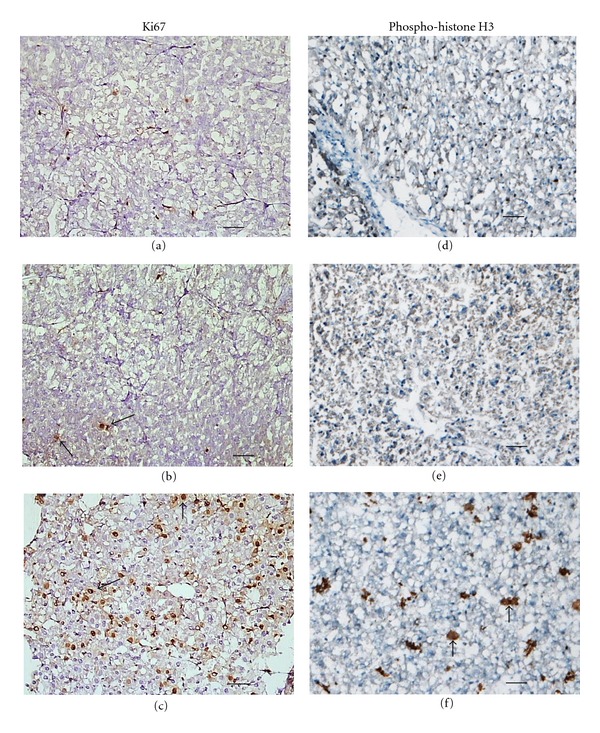
*In situ* immunodetection of Ki67 and phosphohistone H3. *In situ* immunostaining of Ki67 (Figures 7(a–c)) and phosphorylated histone H3 (Figures 7(d–f)) at 48 h of culture in untreated slices (a–d) and after EGF (b–e) or TNF*α* + EGF (c–f) treatments. Arrows indicate Ki67 or phosphohistone H3 positive cells. Bar: 100 *μ*m.

We demonstrated that costimulation by TNF*α* and EGF allow multiple rounds of hepatocyte division in differentiated hepatocytes cocultured with rat liver epithelial cells while the stimulation by EGF alone does induce proliferation [49]. In order to determine if the stimulation with both TNF*α* and EGF triggers DNA replication in cultured liver slices, expression of cyclin D1 and Cdk1 was investigated by immunoblotting in slices stimulated with TNF*α* and EGF (Figure 7(b)). In nonstimulated slices, neither cyclin D1 nor Cdk1 proteins were detected (Figure 6(b)). Similarly, in slices stimulated by EGF or TNF*α*, cyclin D1 was barely detectable despite the expression of P-ERK1 and 2. In EGF-stimulated hepatocytes, used as positive control of proliferation, induction of cyclin D1 protein was observed following EGF stimulation. In contrast, we found that both cyclin D1 and Cdk1 were expressed at 24 and 48 hours of culture in slices stimulated with both EGF and TNF*α*. *In situ* immunodetection of Ki67 (Figures 7(a)–7(c)) and phosphorylated histone H3 (Figures 7(d)–7(f)) in nonstimulated and EGF-stimulated liver slices indicated that very few hepatocytes were stained (<0.5%) in both conditions confirming that hepatocytes in cultured slices did not progress in S phase after stimulation by EGF. However, cotreatment with EGF and TNF*α* induced a strong increase in Ki67 and phosphorylated histone H3 positive cell index reaching ~30% at 48 h (Figure 7(e)).

Altogether, these results demonstrate that hepatocytes had progressed beyond the mitogen-dependent restriction point in mid-G1 phase of the cell cycle in slices stimulated by EGF and TNF*α* and that TNF*α* had primed hepatocytes to allow responsiveness to growth factors.

## 4. Discussion

In normal liver, differentiation and the balance between proliferation and cell death are controlled by complex intercellular communications often referred to as hepatic microenvironment. Alterations of this microenvironment strongly affect differentiation, cell cycle status, and survival. For instance, isolation of hepatocytes by disruption of cell-cell interactions in liver triggers their G0/G1 transition and progression up to mid-G1 phase of the cell cycle [2]. Similarly, *in vitro*, the culture conditions of isolated hepatocytes determine the expression levels of liver specific functions, the capability to proliferate and the cell survival. Hepatocytes cultured in minimal medium are characterized by a rapid decrease in the expression of liver specific functions, the induction of DNA replication upon mitogenic stimulation, a high sensitivity to apoptotic agents, and a 4 to 7 days life-span due to the induction of apoptosis [1]. In contrast, hepatocytes maintained in coculture [10], extracellular matrix sandwiches [12], and Me_2_SO-stimulated cultures [4] are characterized by higher expression levels of liver specific functions maintained for several weeks, the lack of DNA replication upon stimulation by growth factors, and a much higher resistance to apoptotic agents.

The aim of this study was to address the question whether, in liver slices, the integrity of the tissue architecture, and cell-cell communications allowed proliferation of hepatocytes in response to stimulation by a growth factor and protected from apoptosis in culture. Indeed, liver slices provide a unique *in vitro* hepatic model to assess whether the presence of all the liver cell types keeping their cell-cell interactions and polarity affected cell survival and induction of hepatocyte proliferation. Here, we report that cells in liver slices underwent a G0/G1 transition during slicing and progressed up to mid-G1 phase in culture. Indeed, the slicing procedure induced a strong activation of the MEK/ERK, STAT3, and JNK pathways rapidly followed by the transient upregulation of c-fos and constant expression of c-myc mRNA levels, two protooncogenes characterizing the G0/G1, and early G1 phase of the cell cycle, respectively [2]. Concomitantly, expression of GSTA4, a GST isoform induced by lipid peroxydation products and reactive oxygen species, increased as we previously reported during early steps of liver regeneration [41] and during isolation of mouse hepatocytes [50].

Both activation of stress and proliferation signalling pathways and GSTA4 induction most likely result from the cumulated stress signals that occur during the slice preparation procedure including hypoxia, hypothermia, and slicing. This hypothesis is further reinforced by our data evidencing the extracellular LDH release, a decrease in the ATP content, and the transient increase in caspase activities during the first hours of culture. As a consequence, one or two layers of necrotic cells were observed at the center of the slices on the longitudinal histological sections of the liver slices at 24 hours of culture. In addition, the presence of apoptotic bodies was detected at 48 h. These data confirm a postischemic injury following liver perfusion and slicing procedures as previously reported [34, 38] and demonstrate that cell death is heterogeneous within the slices with more necrosis in hypoxic areas. After 24 h, LDH release and caspase activities returned to a basal level, the ATP content went up, and large areas in slices remained viable indicating that the early burst of cell death at 24 h affected only a fraction of cells.

After 24 h of culture, the constant expression of c-myc mRNAs and the induction of p53 mRNAs, two markers of the G1 phase, strongly suggested that cells progressed up to mid-G1 phase. Interestingly, the stimulation by EGF induced phosphorylation of ERK1/2 proteins demonstrating the activation of the MEK/ERK pathway but failed to induce cyclin D1 and Cdk1 expression and DNA replication confirming that hepatocytes in cultured slices were arrested in mid-G1 phase of the cell cycle in presence of growth factor. A large body of evidences demonstrates that liver regeneration following partial hepatectomy is controlled by two groups of extracellular soluble factors [43]. The proinflammatory cytokines TNF*α* and IL-6 are the early stimuli that trigger production reactive oxygen species and redox signalling inducing hepatocyte entry into the cell cycle characterized by the rapid activation of MEK/ERK, STAT3 produced by Kupffer cells and JNK signalling pathways, the preexisting NF*κ*B transcription factor, and the transcriptional induction of a large subset of genes called “immediate-early genes” including c-fos and c-jun [51, 52]. The exit from quiescence and progression in early G1 phase of the cell cycle also called “priming” allows the hepatocytes to become sensitive to growth factors such as HFG, EGF, and TGF*α* that triggers the G1/S transition and the commitment to DNA replication [2, 44].

It is also well-documented that the progression of hepatocytes in late G1 phase during liver regeneration involves the extracellular matrix remodelling [53] and that metalloproteinases MMP-2 and MMP-9 play a crucial role in this remodelling [54]. Similarly, in the coculture model of rat hepatocytes and liver epithelial cells, the induction of the cyclin D1 expression, and the commitment to S phase depends upon the degradation of the extracellular matrix mediated by MMP-9 [49]. Moreover, transcriptional induction of MMP-9 is controlled by TNF*α* establishing a link between this cytokine and extracellular remodelling. 

In cultured slices, the cell cycle arrest in G1 could be due to the maintenance of cell-cell interactions and the absence of extracellular matrix degradation and/or remodelling. Consistently, Vickers et al. [21] recently evidenced an increased expression of collagens in cultured human liver slices that may be linked to activation of stellate cells and/or resident fibroblast. Our data strongly support this hypothesis since the stimulation with both TNF*α* and EGF led to the induction of the cyclin D1, Cdk1, Ki67, and phosphorylated histone H3 demonstrating a progression through S phase and G2/M transition. Our data also suggest that TNF*α* may also be involved in extracellular matrix remodelling in cultured liver slices and future investigations would be required to address this hypothesis. 

Regarding apoptosis, we showed that liver slices maintained in a basal medium did not undergo massive caspase-dependent apoptosis between days 1 and 4 of culture in contrast with high rates of cell death previously reported in pure culture of isolated heptocytes [3, 4]. These data indicate that maintenance of tissue architecture prevented or delayed massive caspase-dependent apoptosis and that loss of cellular integrity observed at 96 h was most likely due to necrosis or other cell death processes that do not involved executioner caspases. Another striking result was the fact that caspase activities and LDH release were moderately induced in cultured slices by apoptotic agents TGF*β*, TNF*α*, and cycloheximide, suggesting that hepatocytes in slices are more resistant to apoptotic agents than isolated hepatocytes in conventional primary culture conditions [55].

However, in liver slices, procaspases 8 and 9 were cleaved into mature forms but their activity remained very low indicating that survival signal(s) blocked the caspase dependent apoptotic pathway beyond the caspase maturation. Similarly, it has been established that Me_2_SO protected hepatocytes from apoptosis in primary culture through the inhibition of both cleaved caspases 8 and 9 and the apoptosis signal-regulating kinase 1 (ASK1), a key element in the cytokine- and stress-induced apoptosis [4]. It was hypothesized that Me_2_SO could inhibit ASK1 activity and the downstream activation of caspases 8 and 9 through preservation of high GST expression levels. Interestingly, while the expression of specific liver proteins such as CYP 3A1/2, 2E1, and albumin, progressively decreased during 4 days of culture, the levels of GSTA1 and P1 remained remarkably stable. It would be interesting to determine whether the high levels of GSTA1/2 detected in cultured slices prevented the activation of ASK1 and caspases 8 and 9.

Altogether, our findings led to the conclusion that hepatocytes in cultured liver slices exhibit a complex phenotype characterized by the reentry into the cell cycle and a G1 phase arrest in absence of appropriate mitogenic stimuli, a robust wave of proliferation following combined stimulation by proinflammatory cytokines and growth factors, and a high resistance to apoptotic stimuli. This latter data reinforce the idea that toxicological data obtained in the models of liver slices may be more accurate and reliable that data obtained in culture of isolated hepatocytes maintained in basal conditions [56]. In addition, the demonstration that hepatocytes in liver slices keep the ability to undergo proliferation opens new perspectives for the use of liver slice in the field of liver regeneration [21] as well as genotoxicity.

## Abbreviations

H: Hours
LDH: Lactate deshydrogenase
CYP: Cytochrome P450
EGF: Epidermal growth factor
ECM: Extracellular matrix
GST: Glutathione S transferase
ASK1: Apoptosis signalling kinase 1
Me_2_SO: Dimethyl sulfoxide
TGF*β*: Transforming growth factor beta
TNF*α*: Tumor necrosis factor.
